# Bridging the Gap: A Holistic View of Personality Factors in Individuals With and Without Alcohol Use

**DOI:** 10.7759/cureus.53935

**Published:** 2024-02-09

**Authors:** Apurva Bezalwar, Pradeep S Patil

**Affiliations:** 1 Psychiatry, Jawaharlal Nehru Medical College, Datta Meghe Institute of Higher Education and Research, Wardha, IND

**Keywords:** prevention and treatment, intervention strategies, protective factors, bidirectional relationship, alcohol use, personality factors

## Abstract

This comprehensive review examines the intricate interplay between personality factors and alcohol use, shedding light on the dynamic relationship that shapes the initiation, progression, and outcomes of alcohol-related behaviors. The exploration encompasses vital personality traits such as sensation seeking, impulsivity, neuroticism, extroversion, agreeableness, conscientiousness, and openness to experience. The bidirectional nature of this association is underscored, emphasizing how personality influences and is influenced by alcohol consumption patterns. Protective personality factors, including resilience, emotional regulation, and social support, are identified as crucial elements in mitigating the risk of alcohol use disorders (AUDs). The implications for clinical practice advocate for tailored interventions that address individual personality profiles, while policy considerations highlight the need for targeted prevention efforts that acknowledge the diverse ways individuals respond to alcohol use. Furthermore, a call for future research emphasizes emerging perspectives, improved methodologies, and ongoing exploration of intervention strategies to advance our understanding of this complex relationship and refine approaches for prevention and treatment. As we navigate this evolving field, the insights gleaned hold promise for shaping more compelling and nuanced interventions to address the diverse needs of individuals affected by AUDs.

## Introduction and background

Understanding the intricate relationship between personality factors and alcohol use has become increasingly crucial in contemporary research. The interplay between an individual's psychological makeup and their propensity for alcohol consumption is complex, influencing the onset, progression, and outcomes of alcohol use disorders (AUDs). The significance of delving into the realm of personality factors lies in its potential to enhance our comprehension of the multifaceted nature of alcohol-related behaviors [1]. The exploration of personality factors in the context of alcohol use provides valuable insights into the predisposing factors that contribute to the initiation and maintenance of problematic drinking behaviors. Individuals vary widely in their responses to alcohol, and these variations are often rooted in diverse personality traits. Investigating these traits not only aids in identifying those at risk for alcohol-related issues but also informs targeted intervention and prevention strategies [2].

Understanding the role of personality factors can shed light on why specific individuals may be more prone to engage in risky drinking behaviors or develop AUDs. In contrast, others may exhibit more controlled patterns of alcohol consumption. This knowledge is essential for developing tailored prevention and treatment approaches that address the unique needs of individuals based on their personality profiles [3]. AUDs represent a spectrum of conditions characterized by problematic alcohol consumption, ranging from mild to severe. These disorders pose significant individual health risks and contribute to societal challenges, including impaired workplace productivity, increased healthcare costs, and social ramifications. The complexity of AUDs necessitates a comprehensive understanding that goes beyond mere biological or environmental perspectives, prompting researchers to investigate the role of personality factors in shaping these disorders [4].

## Review

Personality factors associated with alcohol use

Sensation Seeking

Sensation seeking, as a prominent personality trait, reflects an individual's inherent desire for novel and intense experiences. This characteristic has demonstrated a consistent association with alcohol use, revealing a behavioral inclination toward engaging in stimulating activities. Individuals with high sensation-seeking tendencies are predisposed to seek out experiences that provide excitement and novelty, with alcohol consumption often serving as a means to fulfill this need [5]. The link between sensation seeking and alcohol use is underscored by the observation that individuals scoring high on this trait are more likely to partake in risky behaviors, such as heavy drinking, leading to potential alcohol-related consequences. Recognizing the pivotal role of sensation seeking in influencing alcohol-related behaviors is of paramount importance, as it allows for a targeted understanding of why specific individuals are drawn to the exhilaration associated with alcohol experiences. This understanding, in turn, becomes the cornerstone for tailoring effective prevention and intervention strategies that address the specific needs and risk factors associated with high sensation-seeking individuals. By acknowledging and incorporating the influence of sensation-seeking into preventive efforts, healthcare professionals and policymakers can develop more nuanced and targeted approaches to mitigate the risk of problematic alcohol use among individuals characterized by this trait [6].

Impulsivity

A key personality factor, impulsivity, characterizes the tendency to act on immediate urges without considering potential consequences. This trait has demonstrated a significant and close association with alcohol use, marking it as an influential factor in understanding patterns of drinking behavior. High levels of impulsivity are consistently identified as a risk factor for problematic drinking and the development of AUDs. Individuals exhibiting impulsive tendencies may encounter challenges in inhibitory control, finding it difficult to resist the immediate gratification associated with alcohol consumption [7]. The intricate relationship between impulsivity and alcohol use unveils valuable insights into the cognitive processes that underlie decision-making related to drinking behaviors. Understanding how impulsivity contributes to alcohol use is crucial for the development of targeted interventions, as it addresses the specific challenges impulsive individuals face in regulating their behavior around alcohol. By exploring the nuances of this relationship, researchers and clinicians can refine prevention and treatment strategies that address the cognitive components of impulsivity, ultimately contributing to more effective approaches for mitigating the risk of problematic alcohol use in individuals characterized by this personality trait [8].

Neuroticism

Neuroticism, a personality trait characterized by emotional instability, anxiety, and a propensity to experience negative emotions, emerges as a significant factor in the realm of alcohol use and misuse. Individuals exhibiting high neuroticism scores often turn to alcohol as a coping mechanism, seeking relief from emotional distress and negative affective states. The association between neuroticism and alcohol-related behaviors underscores the intricate interplay between emotional well-being and patterns of alcohol consumption. To address this relationship, it becomes imperative to comprehend how neuroticism contributes to the development and maintenance of problematic drinking behaviors. Unraveling the link between neuroticism and alcohol use is essential for the design of interventions that delve into the emotional underpinnings of drinking, aiming to promote healthier coping mechanisms instead of alcohol reliance. By understanding the role of neuroticism, clinicians and researchers can tailor interventions that address the specific emotional vulnerabilities of individuals with high neuroticism scores, ultimately fostering strategies that diminish the risk of alcohol-related issues and contribute to overall emotional well-being [9].

Extroversion

Extroversion, a personality trait characterized by sociability, assertiveness, and a preference for social interaction, introduces a nuanced dynamic to the landscape of alcohol use. While extroverted individuals may exhibit a greater inclination toward engaging in social drinking, the relationship between extroversion and problematic alcohol use requires careful examination. The impact of extroversion on alcohol consumption patterns extends beyond mere social preferences, offering insights into the intricate social dynamics that shape drinking behaviors. Exploring this association provides a valuable lens through which to understand how extroversion may influence an individual's susceptibility to alcohol-related issues [10]. Extroverted individuals, drawn to social interactions and group settings, may find themselves in environments where alcohol is prevalent, potentially impacting their drinking behaviors. Additionally, the nuanced relationship between extroversion and problematic alcohol use suggests that peer influence and social context play integral roles. Studying the influence of extroversion on alcohol consumption not only deepens our understanding of the social determinants of drinking behaviors but also allows for the identification of specific risk factors associated with this personality trait. This knowledge is critical for informing preventive strategies that account for social dynamics, peer influence, and the unique vulnerabilities of extroverted individuals, ultimately contributing to more targeted interventions in mitigating the risk of problematic alcohol use within this specific personality profile [10].

Agreeableness

Agreeableness, identified by a propensity toward cooperation, trust, and interpersonal warmth, introduces a distinctive perspective on its potential influence on alcohol use, particularly within social relationships. Individuals characterized by high agreeableness may be less inclined to engage in risky drinking behaviors, as their cooperative and accommodating nature tends to foster responsible drinking in social settings. The relationship between agreeableness and alcohol use hinges on the interplay between this personality trait and social dynamics [11]. The cooperative and trusting nature of individuals high in agreeableness can contribute to the establishment of social norms that discourage excessive alcohol consumption. Investigating the role of agreeableness in alcohol use provides valuable insights into how certain personality traits shape the dynamics of social interactions, influencing drinking behaviors within social circles. This exploration contributes to a more comprehensive understanding of how personality factors intersect with social contexts, shedding light on the potential protective influence of agreeableness in mitigating the risk of problematic alcohol use. Recognizing the impact of agreeableness on alcohol-related behaviors is pivotal for tailoring preventive strategies and interventions that leverage the positive social dynamics associated with this personality trait, ultimately contributing to a more nuanced and practical approach to addressing alcohol use within different personality profiles [12].

Conscientiousness

Conscientiousness, defined by self-discipline, goal-directed behavior, and a commitment to rules, establishes a compelling and inverse relationship with alcohol use and misuse, and individuals characterized by high conscientiousness exhibit responsible and controlled drinking patterns, reflecting their disciplined approach to decision-making. In contrast, lower conscientiousness is correlated with an elevated risk of experiencing alcohol-related problems, suggesting a potential vulnerability to impulsive or less-regulated drinking behaviors. Exploring the influence of conscientiousness on drinking behaviors unveils valuable insights into the cognitive and behavioral mechanisms that underpin responsible alcohol consumption [13]. Highly conscientious individuals tend to prioritize self-regulation and goal-oriented decision-making, shaping their approach to alcohol in a controlled manner. Understanding this association is pivotal for designing prevention and intervention strategies tailored to individuals with varying levels of conscientiousness. Emphasizing self-regulation, goal-setting, and adherence to responsible drinking norms can be particularly effective in interventions targeting those with lower conscientiousness. By recognizing the protective nature of conscientiousness against problematic drinking, clinicians and researchers can develop strategies that harness these inherent traits, ultimately contributing to more effective approaches in preventing and addressing alcohol-related issues within diverse personality profiles [14].

Openness to Experience

Openness to experience, delineated by a predilection for novelty, creativity, and a diverse array of experiences, introduces a multifaceted perspective on its association with alcohol-related outcomes. Individuals characterized by high openness may exhibit a greater willingness to experiment with alcohol, reflecting their proclivity for exploring novel and varied experiences. However, the relationship between openness to experience and alcohol use is intricate, influenced by a confluence of other personality factors and contextual elements [15]. The complex interplay of openness to experience with alcohol-related outcomes extends beyond a simplistic positive or negative association. While high levels of openness may contribute to experimental drinking behaviors, the outcomes are nuanced and context-dependent. Investigating the role of openness in alcohol use contributes to a more comprehensive understanding of the diverse motivations and outcomes associated with drinking behavior. This exploration acknowledges that the impact of openness is contingent on various factors, including the presence of other personality traits and the situational context in which alcohol is consumed [16].

Personality differences in individuals with alcohol use

Comparative Analysis of Personality Traits

The association between personality traits and alcohol use has been extensively studied, revealing several vital findings; studies have shown that alcohol consumption correlates positively with sociability and extraversion but negatively with conscientiousness and willingness to conform [17]. Changes in alcohol use were significantly positively associated with changes in aggression-hostility, sensation seeking, and sociability [18]. Personality factors such as emotional stability and vigilance have been linked to the severity of alcohol use, with emotional stability and vigilance being associated with greater severity of drinking [19]. A study from the United Kingdom found that personality traits, such as aggression-hostility, sensation seeking, and sociability, were associated with the frequency of alcohol intoxication in young males and females [20]. Personality traits play a significant role in alcohol use, with various traits influencing drinking behavior and the development of AUDs. Understanding these associations is crucial for developing effective interventions and treatments for individuals with alcohol-related issues.

Subgroup Variations

The association between personality traits and alcohol use varies across different subgroups, such as gender, age, and cultural influences. A study conducted among young Swiss men found that changes in alcohol use were significantly positively associated with changes in aggression-hostility, sensation seeking, and sociability [18]. Another study from the United Kingdom explored the associations between personality traits and the frequency of alcohol intoxication in young males and females, indicating that personality traits play a role in alcohol drinking and intoxication [20]. Research has highlighted that normative changes may impact personality across different age segments, such as adolescents, young adults, and older individuals [18]. Cultural factors may also influence the relationship between personality traits and alcohol use. However, specific studies focusing on the interaction between cultural influences and personality traits in the context of alcohol use were not found in the provided search results [15]. The association between personality traits and alcohol use can be influenced by various factors, including gender, age, and potentially cultural influences. Further research is needed to comprehensively understand the subgroup variations in the relationship between personality traits and alcohol use.

The interplay between personality and alcohol use

Bidirectional Relationship

The interplay between personality and alcohol use is characterized by a bidirectional relationship, where personality traits influence alcohol-related behaviors. A meta-analysis of 72949 adults from eight cohort studies found that heavy alcohol consumption was associated with higher extraversion and neuroticism and lower agreeableness [15]. Conversely, alcohol use may shape or exacerbate specific personality characteristics. Understanding this dynamic relationship is essential for unraveling the complex mechanisms that contribute to the initiation, maintenance, and escalation of AUDs. Research suggests that certain personality traits may predispose individuals to alcohol use, while the experience of alcohol-related consequences can, in turn, influence the development or modification of personality traits. This bidirectional relationship underscores the need for comprehensive longitudinal studies to disentangle the intricate connections between personality and alcohol use over time [21].

The Role of Stress and Coping Mechanisms

Stress and coping mechanisms play a pivotal role in the interplay between personality and alcohol use. Individuals with specific personality traits, such as high neuroticism, may be more susceptible to stressors, prompting them to turn to alcohol as a coping mechanism. Understanding how personality factors contribute to stress vulnerability and how individuals choose to cope through alcohol use provides insights into the motivational aspects of drinking behavior. Moreover, examining the effectiveness of various coping strategies within different personality profiles offers valuable information for tailoring interventions that address the underlying stressors driving alcohol consumption [22].

Genetic and Environmental Influences

A combination of genetic and environmental factors further influences the interplay between personality and alcohol use. Genetic predispositions may contribute to both personality traits and susceptibility to AUDs. Family and environmental influences, such as parental modeling of drinking behavior and social norms surrounding alcohol use, also shape an individual's personality and attitudes toward alcohol. Investigating the complex interplay of genetic and environmental factors elucidates the pathways through which personality traits and alcohol use interact. This understanding is crucial for developing targeted prevention and treatment strategies that consider the unique genetic and environmental contexts influencing the relationship between personality and alcohol use [23]. The integration of these three components - the bidirectional relationship, the role of stress and coping mechanisms, and genetic and environmental influences - provides a comprehensive framework for comprehending the intricate interplay between personality and alcohol use. Recognizing the multifaceted nature of these interactions is essential for informing evidence-based interventions that address the underlying mechanisms driving alcohol-related behaviors in individuals with diverse personality profiles [24].

Protective personality factors

Resilience

Resilience, the ability to adapt positively in the face of adversity, emerges as a critical protective factor against the development of problematic alcohol use. Individuals characterized by higher levels of resilience exhibit an enhanced capacity to cope with life stressors, reducing their susceptibility to turning to alcohol as a maladaptive coping mechanism. Exploring the role of resilience in the context of alcohol use provides insights into the mechanisms by which individuals can navigate challenges without resorting to excessive or harmful drinking behaviors. Understanding resilience as a protective factor informs interventions aimed at bolstering individuals' ability to withstand stressors and adversity, ultimately reducing the risk of alcohol-related problems [25].

Emotional Regulation

Emotional regulation, the ability to manage and modulate one's emotional responses, plays a crucial role in protecting against problematic alcohol use. Individuals with practical emotional regulation skills are better equipped to navigate stressors without relying on alcohol as a means of emotional escape. Examining the relationship between emotional regulation and alcohol use provides valuable insights into how the regulation of affective states may mitigate the risk of developing alcohol-related issues. Interventions targeting emotional regulation skills offer a promising avenue for preventing and addressing AUDs by enhancing individuals' adaptive coping strategies [26].

Social Support

Social support, encompassing emotional, instrumental, and informational assistance from social networks, serves as a potent protective factor against problematic alcohol use. Robust social support systems provide individuals with alternative coping mechanisms and reduce the likelihood of turning to alcohol as a solitary coping strategy. Investigating the role of social support in the context of personality and alcohol use sheds light on the interpersonal dynamics that influence drinking behaviors [27]. Moreover, understanding how social support interacts with personality traits offers insights into the nuanced ways in which social connections contribute to protective outcomes. Incorporating social support into prevention and intervention efforts can enhance the effectiveness of strategies aimed at mitigating the impact of personality factors on alcohol use [27].

Intervention strategies

Personality-Targeted Interventions

Personality-targeted interventions represent a promising approach to addressing AUDs by tailoring strategies to individuals' specific personality profiles. These interventions recognize that different personality traits may contribute to the initiation and maintenance of problematic drinking behaviors. Researchers and clinicians can create more effective and targeted approaches by customizing interventions based on personality characteristics. Understanding how specific personality traits interact with intervention strategies allows for the development of personalized and adaptive programs that address the unique needs of individuals. Exploring the efficacy of personality-targeted interventions contributes to a growing body of evidence supporting the importance of individualized approaches in preventing and treating AUDs [28].

Psychoeducation

Psychoeducation interventions focus on providing individuals with information and skills related to alcohol use, its consequences, and healthy coping mechanisms. This approach aims to enhance individuals' knowledge about the interplay between personality and alcohol use, fostering a deeper understanding of the factors that contribute to problematic drinking behaviors. Psychoeducation interventions may include components that target risk and protective factors associated with specific personality traits. Investigating the impact of psychoeducation on individuals with diverse personality profiles informs the development of educational programs that address the unique needs and vulnerabilities of different populations. By empowering individuals with knowledge and skills, psychoeducation interventions contribute to the prevention and reduction of alcohol-related problems [29].

Cognitive-Behavioral Therapy

Cognitive-behavioral therapy (CBT) represents a well-established and effective therapeutic approach for individuals with AUD. CBT aims to modify maladaptive thoughts and behaviors associated with alcohol use by addressing cognitive distortions and developing healthier coping strategies. In the context of personality and alcohol use, CBT can be tailored to target specific personality traits influencing drinking behaviors. For example, interventions may focus on enhancing impulse control, managing stressors, or improving emotional regulation. Examining the effectiveness of CBT in individuals with different personality profiles contributes to refining therapeutic techniques that align with the unique challenges posed by various traits. CBT's adaptability makes it a valuable tool in addressing the intricate interplay between personality factors and alcohol use [30].

Future directions in research

Emerging Perspectives on Personality and Alcohol Use

Neurobiological correlates: As we witness ongoing technological advancements in neuroscience, future research should delve into the neurobiological foundations that underlie the intricate relationship between personality traits and alcohol use. Employing cutting-edge technologies and methodologies, investigations can explore how specific personality traits are intricately linked to neural processes and neurotransmitter systems. Unraveling these neurobiological correlates can furnish a more holistic understanding of the biological mechanisms steering alcohol-related behaviors. This avenue of research has the potential to not only elucidate the neural basis of the interplay between personality and alcohol use but also to identify potential targets for novel therapeutic interventions aimed at mitigating the risk of problematic drinking [31].

Cross-cultural studies: An emerging and crucial area for future research involves examining how personality factors influence alcohol use across diverse cultural contexts. Cultural variations in the expression of personality traits and drinking norms may interact in complex ways, shaping the risk and protective factors associated with alcohol use. By conducting cross-cultural studies, researchers can contribute to a more nuanced understanding of the cultural dimensions that intricately shape the interplay between personality and alcohol use. This knowledge is vital for tailoring prevention and intervention strategies that respect and address the cultural intricacies influencing drinking behaviors, ultimately ensuring the effectiveness of these approaches across diverse populations [32].

Developmental trajectories: Longitudinal studies focusing on the developmental trajectories of personality and alcohol use, spanning from adolescence into adulthood, stand as a pivotal avenue for future research. Tracking how personality evolves and understanding its impact on alcohol-related behaviors at different life stages provides valuable insights. This research approach enables the identification of critical periods during development where certain personality traits may pose a higher risk for problematic drinking. The findings from such studies can inform targeted prevention efforts, allowing for interventions tailored to specific developmental periods. By considering the dynamic nature of personality and its evolution across the lifespan, researchers can develop more effective strategies for preventing and addressing alcohol-related issues at various stages of life [33].

Methodological Improvements in Studying Personality Factors

Ecological momentary assessment: Embracing ecological momentary assessment (EMA) methodologies represents a pivotal advancement in research design, enabling real-time capture of fluctuations in both personality and alcohol use. By incorporating EMA, researchers can transcend the limitations of retrospective reporting, gaining access to moment-to-moment variations in behavior and personality traits. This approach enhances the ecological validity of research findings by exploring how personality manifests in daily life and its immediate impact on drinking behaviors. EMA's ability to capture real-world experiences offers a nuanced understanding of the temporal dynamics between personality traits and alcohol use, ultimately contributing to a more accurate and contextually relevant portrayal of these complex interactions [34].

Multi-method assessments: Integrating multiple assessment methods is a methodological imperative for advancing our understanding of how personality factors contribute to alcohol use. Beyond relying solely on self-report measures, researchers should incorporate diverse approaches, including behavioral observations and physiological markers. This multi-method approach strengthens the validity and reliability of personality assessments by triangulating information from various sources. Behavioral observations provide an objective lens into actual behaviors, while physiological markers offer insights into the biological underpinnings of personality traits. By combining these diverse data sources, researchers can construct a more comprehensive and nuanced understanding of the intricate relationships between personality and alcohol use, mitigating potential biases associated with relying on a single assessment method [35].

Big data analytics: The era of big data analytics and machine learning presents an unparalleled opportunity to unravel complex patterns within extensive datasets related to personality and alcohol use. Leveraging these advanced analytical techniques enables researchers to identify subtle interactions and associations that traditional analyses might overlook. Analyzing vast datasets from diverse populations allows for the identification of nuanced relationships between personality factors and alcohol use, shedding light on the heterogeneity within these associations. The use of big data analytics not only facilitates the exploration of intricate patterns but also allows for the development of predictive models that can enhance our understanding of the multifaceted interplay between personality and alcohol use. As technology evolves, embracing big data analytics is a transformative approach to uncovering novel insights and refining our understanding of these complex relationships [36].

Implications for Prevention and Treatment

Tailored interventions: The future landscape of research should prioritize the refinement and expansion of personality-tailored interventions for AUDs. Researchers can enhance the precision and effectiveness of prevention and treatment strategies by honing in on specific personality profiles that exhibit differential responses to interventions. Tailored interventions acknowledge the heterogeneity within populations, recognizing that a one-size-fits-all approach may not be optimal. Identifying specific personality traits that respond favorably to certain interventions enables the development of targeted and personalized approaches, ultimately optimizing outcomes and reducing the risk of relapse [37].

Integration of technology: Integrating technology, including mobile applications and virtual platforms, represents a promising frontier in enhancing the accessibility and engagement of prevention and treatment programs for AUDs. Technology-driven interventions offer a dynamic and interactive means of reaching individuals with diverse personality profiles. By leveraging mobile applications and virtual platforms, interventions can be personalized to target specific personality traits, providing tailored content and support. The accessibility and flexibility afforded by technology-driven approaches contribute to improved engagement, adherence, and the long-term effectiveness of interventions. Exploring technology integration aligns with individuals' evolving preferences and lifestyles, ensuring that interventions remain relevant, engaging, and impactful [38].

Translational research: Bridging the gap between research findings and clinical practice through translational research is paramount in personality-informed prevention and treatment approaches. This involves developing evidence-based guidelines that seamlessly translate research insights into practical and applicable strategies for clinicians and policymakers. Translational research ensures that the valuable knowledge generated from research efforts directly informs real-world practices, optimizing the impact of personality-informed interventions. By facilitating the seamless transition of evidence-based approaches into clinical settings, translational research contributes to integrating cutting-edge findings into routine practice, ultimately improving the delivery and efficacy of prevention and treatment programs for AUDs [39].

## Conclusions

In conclusion, this comprehensive review underscores the intricate relationship between personality factors and alcohol use, revealing the profound influence of traits such as sensation seeking, impulsivity, and neuroticism on the initiation and progression of alcohol-related behaviors. The bidirectional nature of this association highlights the dynamic interplay between personality and alcohol use, emphasizing how one's psychological makeup both shapes and is shaped by patterns of alcohol consumption. Protective factors, including resilience, emotional regulation, and social support, emerge as critical elements mitigating the risk of AUD. These findings directly impact clinical practice, advocating for tailored interventions that address individual personality profiles, incorporate psychoeducation, and integrate cognitive-behavioral therapies. On a policy level, recognizing the role of personality factors calls for a shift toward more targeted prevention efforts that acknowledge the diverse ways in which individuals respond to alcohol use. The call for further research in emerging perspectives, improved methodologies, and ongoing exploration of intervention strategies is essential for advancing our understanding of this complex relationship and refining approaches for prevention and treatment. As we continue to unravel the complexities of personality and alcohol use, this evolving field holds promise for shaping more compelling and nuanced interventions to address the diverse needs of individuals affected by AUD.
